# Using a GIS to support the spatial reorganization of outpatient care services delivery in Italy

**DOI:** 10.1186/s12913-018-3642-4

**Published:** 2018-11-22

**Authors:** Martina Calovi, Chiara Seghieri

**Affiliations:** 1 Geoinformatics and Earth Observation Laboratory, Department of Geography and Institute for CyberScience, The Pennsylvania State University, University Park, PA USA; 2 Management and Healthcare Lab, Institute of Management, Sant’Anna School of Advanced Studies, Piazza Martiri della Libertà, 24, 56127 Pisa, Italy

**Keywords:** Health care management, GIS, Two-step floating catchment area method, Spatial accessibility, Outpatient; closure simulation

## Abstract

**Background:**

Studying and measuring accessibility to care services has become a major concern for health care management, particularly since the global financial collapse. This study focuses on Tuscany, an Italian region, which is re-organizing its inpatient and outpatient systems in line with new government regulations. The principal aim of the paper is to illustrate the application of GIS methods with real-world scenarios to provide support to evidence-based planning and resource allocation in healthcare.

**Methods:**

Spatial statistics and geographical analyses were used to provide health care policy makers with a real scenario of accessibility to outpatient clinics. Measures for a geographical potential spatial accessibility index using the two-step floating catchment area method for outpatient services in 2015 were calculated and used to simulate the rationalization and reorganization of outpatient services. Parameters including the distance to outpatient clinics and volumes of activity were taken into account.

**Results:**

The spatial accessibility index and the simulation of reorganization in outpatient care delivery are presented through three cases, which highlight three different managerial strategies. The results revealed the municipalities where health policy makers could consider a new spatial location, a shutdown or combining selected outpatient clinics while ensuring equitable access to services.

**Conclusions:**

A GIS-based approach was designed to provide support to healthcare management and policy makers in defining evidence-based actions to guide the reorganization of a regional health care delivery system.

The analysis provides an example of how GIS methods can be applied to an integrated framework of administrative health care and geographical data as a valuable instrument to improve the efficiency of healthcare service delivery, in relation to the population’s needs.

## Background

To address the financial pressure resulting from the economic crisis in Europe (2008-date), and with the growing proportion of the elderly in the population (24% aged 60 or over) [1], policy makers are increasingly focusing on finding an acceptable balance between adequate cost control measures and ensuring that the population continues to receive high-quality, appropriate and efficient care [2].

The European Observatory suggests that policy makers should ascertain which tools respond best to the needs and consider the impact of the reforms they propose to achieve better health system goals [2]. These include financial protection, efficiency, equity, quality, responsiveness, transparency, and accountability [2, 3].

The Italian national health system follows the Beveridge model [4, 5] and provides universal coverage for comprehensive and essential health services through general taxation, recognizing health as a fundamental right of individuals and of collective interest for society. In the early 1990s, national reforms were implemented to transfer several key administrative and organizational responsibilities from central government to the 20 Italian regional administrations, thus making regional authorities more sensitive to controlling expenditure and promoting efficiency, quality, and patient satisfaction. The regional administrations are now responsible for organizing and delivering health services through local health units [6].

Like other EU countries, Italy has taken legislative measures and implemented a set of policy initiatives to respond to the economic crisis, through agreements between the national and regional governments, health pacts, and through annual finance acts and other legislative measures to control public spending [6]. The overall aim is to rationalize and to reorganize the health services by defining quality, structural, and technological standards. These include the expected catchments areas for each care discipline, and volume thresholds for specific surgical procedures (Health Minister Decree 20 April 2015). Regional governments are therefore attempting to balance the need for national standards, while providing an equitable geographical resource distribution.

Thus, the main objective of this research is to provide evidence on how a geographical information system (GIS) can be used to support health care management in the reorganization of care service delivery, by computing and analyzing spatial access to outpatient services relative to the demand.

GIS facilitates a unique geographic approach to a variety of problems by assessing the current distribution of necessary resources and anticipated future needs. As most health phenomena can be described spatially, GIS can be effectively used as a decision support system, not only in health care management but also in addressing urban planning, transportation, energy, and resource analyses. GIS technologies highlight the relationships between different kinds of data, enabling complex data to be interpreted in an easier and more realistic manner [7].

GIS has become essential in the health and healthcare sector in recent years. Extensive literature examines the use of GIS technology in epidemiology, and typically analyzes the relationships between locations, environments, and diseases, studing their distribution and monitoring their dissemination over space and time [8].

Digital health care data have led to much interest in geospatial analyses in the healthcare management research field. Hilton et al. [9] outlined in 2005 a combination of GIS and human health applications within decision-making, using Anthony’s Model [10], and defined this approach as “the management of people, assets, and services using spatial information to ensure the delivery of the health care service while assuring that specific tasks are carried out effectively and efficiently” [9].

The understanding of the spatial distribution of services is important for the improvement of the efficiency of care delivery organizations, while providing citizens with healthcare services that optimally respond to their needs. Creating an integrated framework of administrative health care and geographical data enables planners and managers to visualize and examine the impact of government policy decisions [11] over the territory. In particular, GIS approaches, spatial statistics analyses, and the measurement of accessibility indexes can be useful to inform policy makers on locating population needs, understanding where they are not covered by the local supply of services [12], or where there may be an oversupply.

Indeed, studying and measuring access to care services has been a major concern for health care management since the early 1970s [13–20]. Today, the importance of measuring spatial access to care services is well known and, as Guatam et al. stated in 2014, these kinds of measurements are important instruments that can help care managers provide more respondent services and to reduce spatial inequalities [21]. The importance of a spatial analysis has thus been established. Gautam et al. noted in 2014 that in the U.S. “effectively evaluating spatial healthcare disparity is crucial to improve health care access” [21].

In this context, the current distribution of the outpatient services in the Tuscany region (Italy) is analyzed, in relation to the population and to the current demand for services, using a comprehensive geographical health information infrastructure. The aim is to support the regional administration and care managers in terms of improvements in governance and equitable outpatient service delivery.

More broadly, our study addresses the question “how can an integrated framework of administrative health care and geographical data support the health care management in the reorganization, redesign and planning of health care services?”

The two-step floating catchment area (2SFCA) method is used to define the spatial access to outpatient services, based on the availability and accessibility of the outpatient facilities, and to examine access to the current service use in Tuscany in 2015. Oversupplies or lack of supplies are identified based on spatial criteria. This knowledge can help reorganize the care services delivery system more efficiently, and ensure universal and equitable access for the entire population.

The paper is divided into five sections. Section “BACKGROUND” outlines the methodology, presenting the spatial distribution of the outpatient clinics and the two-step floating catchment area method. Section “METHODS” outlines the data used, the demand, the supply, and the unit of analysis. Section “RESULTS” provides the results and presents the closure simulation. Section “DISCUSSION” outlines the discussion and the last section “CONCLUSIONS” presents the conclusions.

## Methods

Spatial accessibility can be regarded as the balance between supply and demand within a geographical area, and we chose the 2SFCA method to compute the potential spatial accessibility index [22, 23]. The integrated analysis of this method combines distances and the supply-demand ratio, and is considered one of the best methods to measure potential spatial accessibility to health care services, through establishing the geographical location of services based on population needs [12, 24, 25].

The framework proposed in this study consists of three stages. First, the potential spatial accessibility index is implemented. Second, the potential reorganization of the outpatient services is simulated based on the results of the first step, and a new management solution is then suggested. The third and last step consists of computing a new potential spatial accessibility index to verify the repercussions of the managerial strategies throughout the outpatient system.

This integrated geographical framework combines administrative health care and geographical data, and can help health care planners visualize the impact of their policies.

### Data

The 2SFCA method requires the key data elements of population data (demand), healthcare service locations, and volumes data (supply), and the measured distance between demand and supply.

The data used for the analysis include individual-level administrative care data on all residents of Tuscany. An anonymous ID is assigned to each patient, which enables residents in Tuscany to be tracked in terms of access and use of any healthcare services. A regional administrative health care dataset of outpatient specialized visits in 2015 has been used in this research.

From the original dataset, the main clinical disciplines that patients could access were extracted: cardiology, gynecology, neurology, orthopedics, otorhinolaryngology, ophthalmology, dermatology, urology, and general surgery. Consultations provided at ERs have not been included.

All the statistical analyses were run using SAS version 9.4 (SAS Institute), and the geographical analyses were run using ArcMap version 10.3.1 (ESRI).

### Unit of analysis

**Fig. 1:**
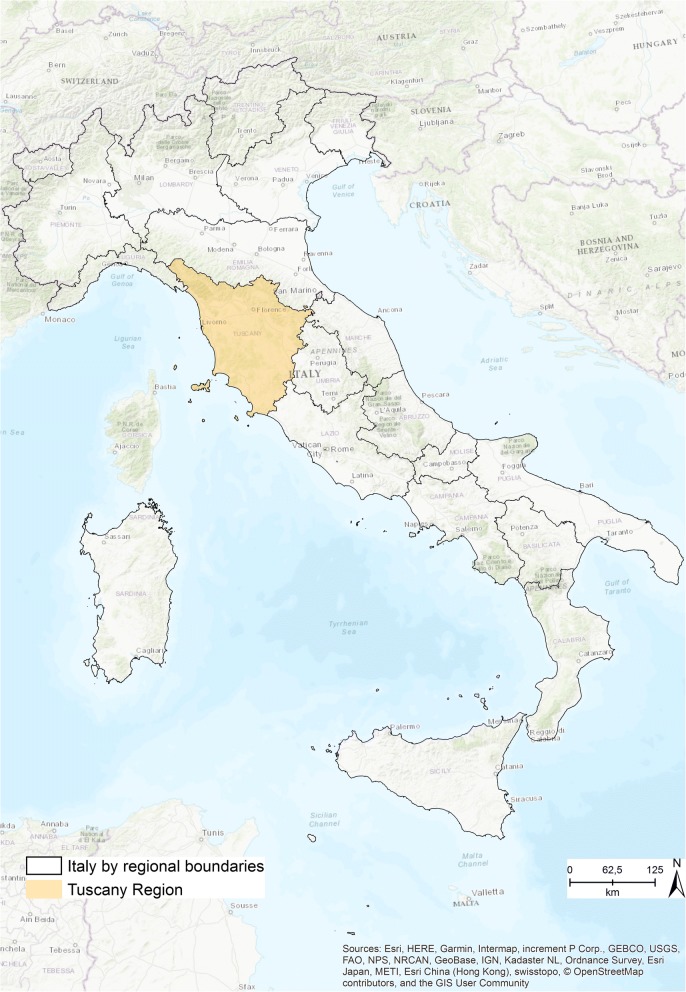
Italy and Tuscany Region [The figure is created by the authors and is not taken from other sources]

The study focuses on Tuscany, an Italian central region (Fig. 1). The Tuscan regional administration is subdivided into 280 municipalities that belong to 10 provinces. Since January 1, 2016, regional law 84/2015 splits the regional healthcare system into three new local health authorities (LHAs), which combine four teaching hospitals and the 12 previous LHAs.

The overall territory is divided into three “large areas” and 25 health districts, which are in charge of organizing and delivering the services of territorial health networks, social care, and social integration.

All the analyses were run at the municipality level, which is the smallest administrative unit, as the scale of analysis in the study is the smallest level of the territory.

### Demand

According to the administrative health care database, Tuscany has more than 3.7 million inhabitants, ranging from the smallest municipality of the island of Capraia with 416 citizens, to the largest of Florence with 381,037 inhabitants (Table 1). For each municipality, a georeferenced centroid was defined, combining the residents of each municipality into a corresponding single point.

**Table 1:** – Population of Tuscany: descriptive statistics

Variable	Municipality	Total Population	Male	Female
N	280	3752.654	1,804,558	1,946,676
Mean		13,402.34	6444.85	6952.41
Std dev.		30,264.38	14,301.83	15,966.70
Min		416	228	188
Max		381,037	178,214	202,823

The dasymetric mapping methodology was used to create the population centroids by interpolating residential land use areas, as available on the website of the Tuscan regional administration [26, 27]. The lack of public information on single individual residence addresses prompted this approach, and the methodology was chosen as it enables the distribution of the aggregated population data within each unit of analysis to be better estimated.

### Supply

The selection criteria used to analyze the original dataset revealed that there were 246 outpatient clinics throughout Tuscany in 2015, which were accessed by patients more than two million times. Each clinic was integrated into a GIS environment, by geolocating the clinics by their addresses over the 280 municipalities of Tuscany. Of these municipalities, 120 have no outpatient clinics under their jurisdiction.

The total number of visits provided by each outpatient clinic for this study represented the health services delivery for each municipality, which range from 106 to 145,956. On average, the 246 outpatient clinics provided 8358 specialized visits in 2015.

### Travel distance

Studies on accessibility either use Euclidean/linear distance or travel time distance. The Tuscan regional road network, available on the Open Toscana website, was used to calculate the travel time distances. The regional road network dataset is composed of linear elements (arches) that represent all the segments of each road and that are defined by points (nodes) at the junctions. The original dataset has much missing data. To avoid errors during the analyses, information on travel speed and road type have been interpolated, to fill in all the missing records and obtain a complete dataset. To calculate the distances between the centroids of the municipalities (origins) and the outpatient clinics (destinations), an OD matrix was computed. The mean distance is about 73 min, the minimum distance is equal to a few minutes, and the maximum distance is about 3 h.

### Two-step floating catchment area method

The 2SFCA method, based on the spatial decomposition concept of Radke and Mu [28], has been chosen to conduct the potential spatial accessibility index. This methodology is selected instead of a more sophisticated gravity model because the results are easily readable and intuitive, and because the algorithm overcomes cross-border limitations [20, 22, 29]. The research aim was to run all the analyses at the most local level to obtain results that best fit the needs of the population’s health services. According to this bases and to the analysis level, the threshold of the catchment areas has been set at a travel time of 15 min [21].

In this analysis, supply is represented by the specialized visits supplied by each clinic, while the total resident population for each municipality represents the demand.

A conventional 2SFCA model was used. For each supply location, *j*, the first step detects all demand locations, *k*, that are within a threshold time distance *d0* from *j*, and then computes the supply-to demand ratio R*j* for the inhabitants within the catchment area:

$$ R\mathrm{j}=\frac{S\mathrm{j}}{\sum_{k\in \left\{d\mathrm{kj}\le d0\right\}}\frac{D\mathrm{k}}{1000}} $$

Where *d*_*kj*_ is the distance between *k* and *j, D*_*k*_ is the demand at location *k* that falls within the catchment, and finally S_*j*_ is the capacity of supply [23].

The second step detects each demand location *I* and all supply locations *j* within the threshold time distance *d*0 from location *I,* and adds together the supply-to-demand ratios *R*_*j*_ at those locations, to obtain the full accessibility *Ai F* at demand location *i*.

$$ {A}_i^F=\sum \limits_{j\in \left\{d\mathrm{ij}\le d0\right\}}R\mathrm{j}=\sum \limits_{j\in \left\{d\mathrm{ij}\le d0\right\}}\left(\frac{S\mathrm{j}}{\sum_{k\in \left\{d\mathrm{kj}\le d0\right\}}\frac{D\mathrm{k}}{1000}}\right) $$

Where *d*_*ij*_ is the distance between *i* and *j*, and *R*_*j*_ is the supply-to-demand ratio at supply location *j* that falls within the catchment centered at i [22].

### Reorganization simulation

The potential spatial accessibility index detects the dynamics of the spatial access to the outpatient clinics, while the reorganization simulation is useful to assess the effects of the possible strategies for restructuring the regional outpatient service network. The simulation focuses on the municipalities with a high potential accessibility index, equal to or over .65. These represent the municipalities in which residents have access to a minimum of .65 specialized visits within 15 min [12]. Each municipality was analyzed in depth, creating specific scenarios to study the supply, demand, and the travel time distances. The clinics from municipalities characterized by high accessibility were subdivided into quintiles of yearly volume of outpatient visits. The units that were part of the first quintile class (low-volume clinics) were considered in the closure simulation. This threshold has been fixed because, to our knowledge, there are no specific standards that identify any minimum threshold for the number of outpatient visits that should be provided in a year.

For each scenario, a strategic solution was proposed that guarantees accessibility to the clinics within the minimum possible time, thus maintaining an efficient service provision. All the simulated managerial solutions focus on reorganizing the distribution of the clinics in municipalities where an oversupply was identified.

The next step involved running a new potential spatial accessibility index based on the updated number of clinics resulting from the closure simulation. The findings, and the differences between the initial and second indexes performed, highlight the repercussions stemming from a clinics’ potential closure and can verify if equitable accessibility has been guaranteed to all patients.

## Results

To better understand the spatial distribution of the outpatients’ clinics, various spatial statistical analyses were run as exploratory tests. The nearest neighbor index was run to measure the degree of spatial dispersion in the distribution based on the minimum inter-feature distances, and the weighted standard deviational ellipse was then computed to check the distribution of these features [12].

**Fig. 2:**
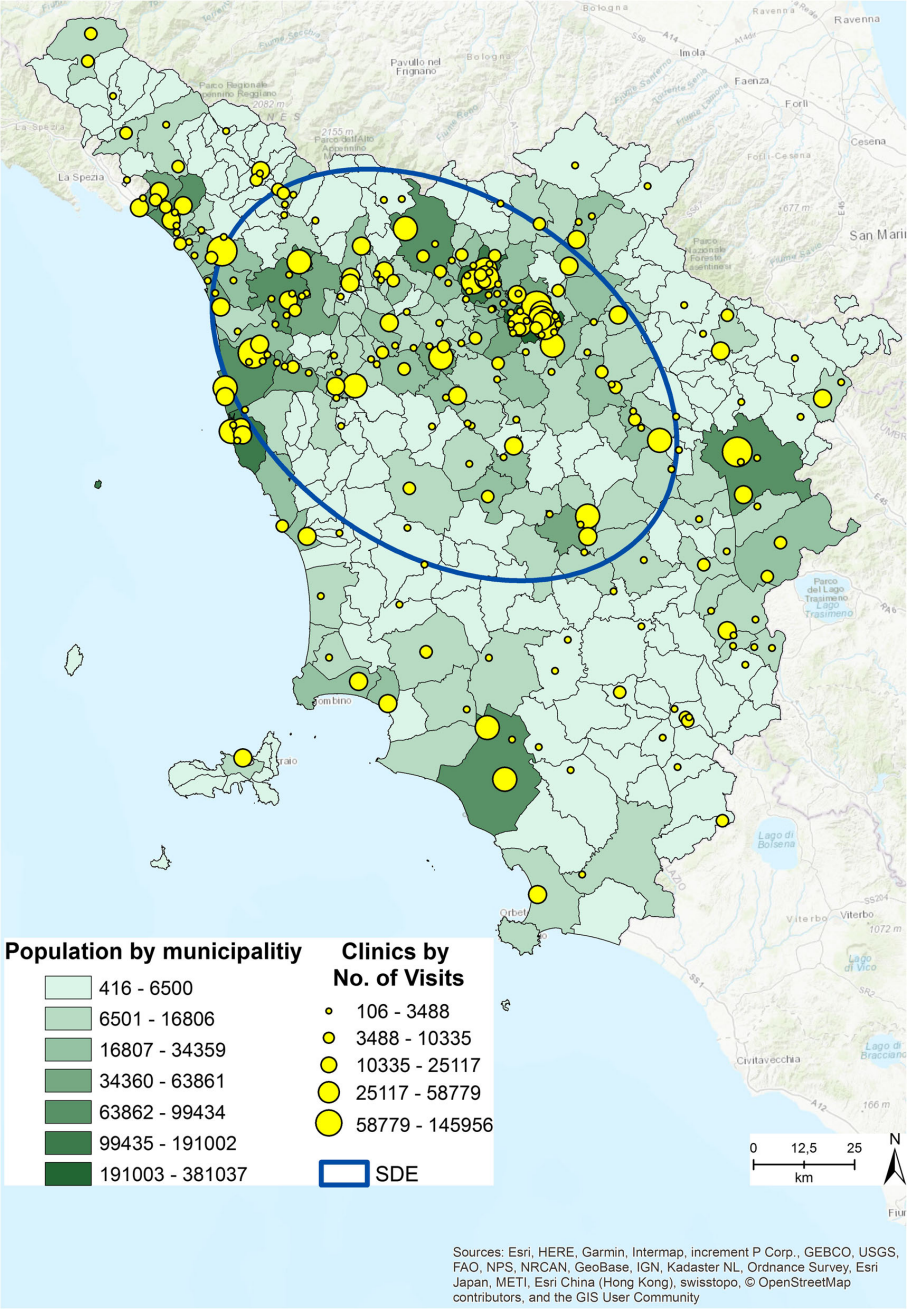
Population distribution by municipalities, Clinics distribution by No. of Visits, SDE [The figure is created by the authors and is not taken from other sources]

The average nearest neighbor returned a ratio between the observed average distance and the expected average distance based on a hypothetical random distribution of the same sample features over the same area. The average distance ratio typically varies from 1, representing high clustering to − 1, meaning that the objects are sparse in the geographical area. Our pattern value was 0.99, and thus the pattern exhibits clustering and a high concentration of clinics in this area. The average distance between two outpatient clinics, given by the expected mean distance, was 6 km [30]. The directional distribution returned the standard deviational ellipse (SDE) (Fig. 2) based on the locations of the clinics and the total number of outpatient visits (ESRI report) as the associated attribute value. The directional trend confirmed the cluster area identified using the point pattern statistics. These techniques enabled us to better understand the regional distribution of the outpatient clinics.

Figure 2 shows the distribution of the regional population by municipalities, the distribution of the clinics by number of visits provided, and the SDE. The map suggests a correlation between the two distributions. As Smith et al. [31] highlighted, understanding the spatial distribution of a population is important to ensure adequate spatial accessibility to healthcare services [31].

**Fig. 3:**
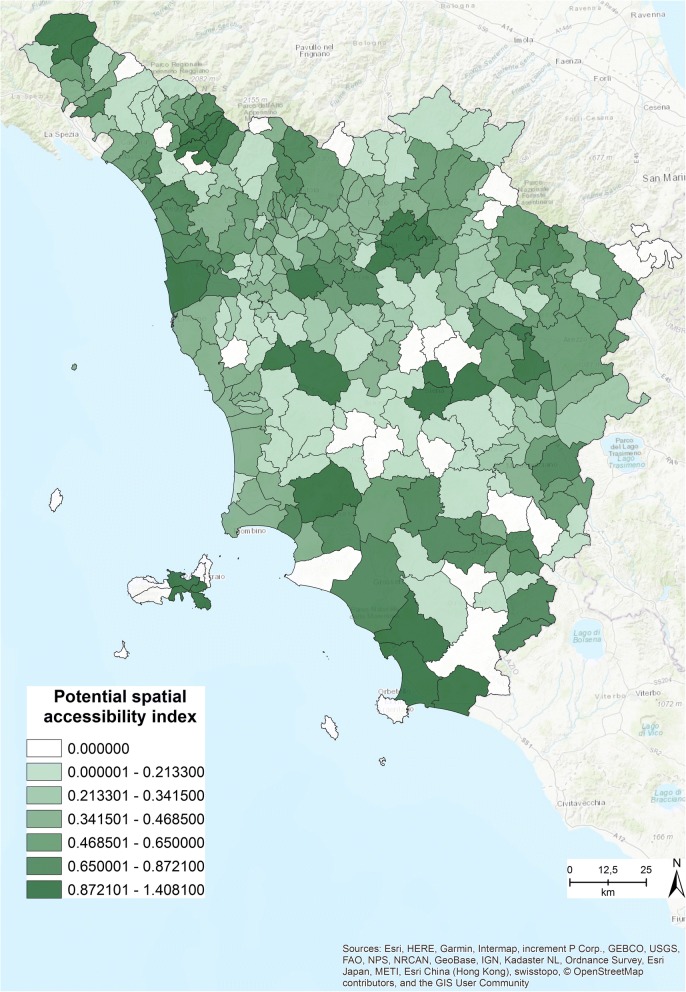
Potential spatial accessibility index within 15 min [The figure is created by the authors and is not taken from other sources]

Figure 3 shows the potential spatial accessibility index, obtained by running the 2SFCA method. The index ranges from .0001 to 1.408, with an average of .415 specialized visits for each citizen within in 15 min’ drive [12] and a standard deviation of .325.

Each value of the index refers to the number of specialized clinics that each person has access to in 15 min within the municipality he or she lives in. Those who live in the municipality with the highest value have access to 1.408 specialized visits within 15 min’ drive [12]. In Fig. 3 the shades of green correspond to the range of the index. The light colors refer to low accessibility values and the dark colors to high values.

**Fig. 4:**
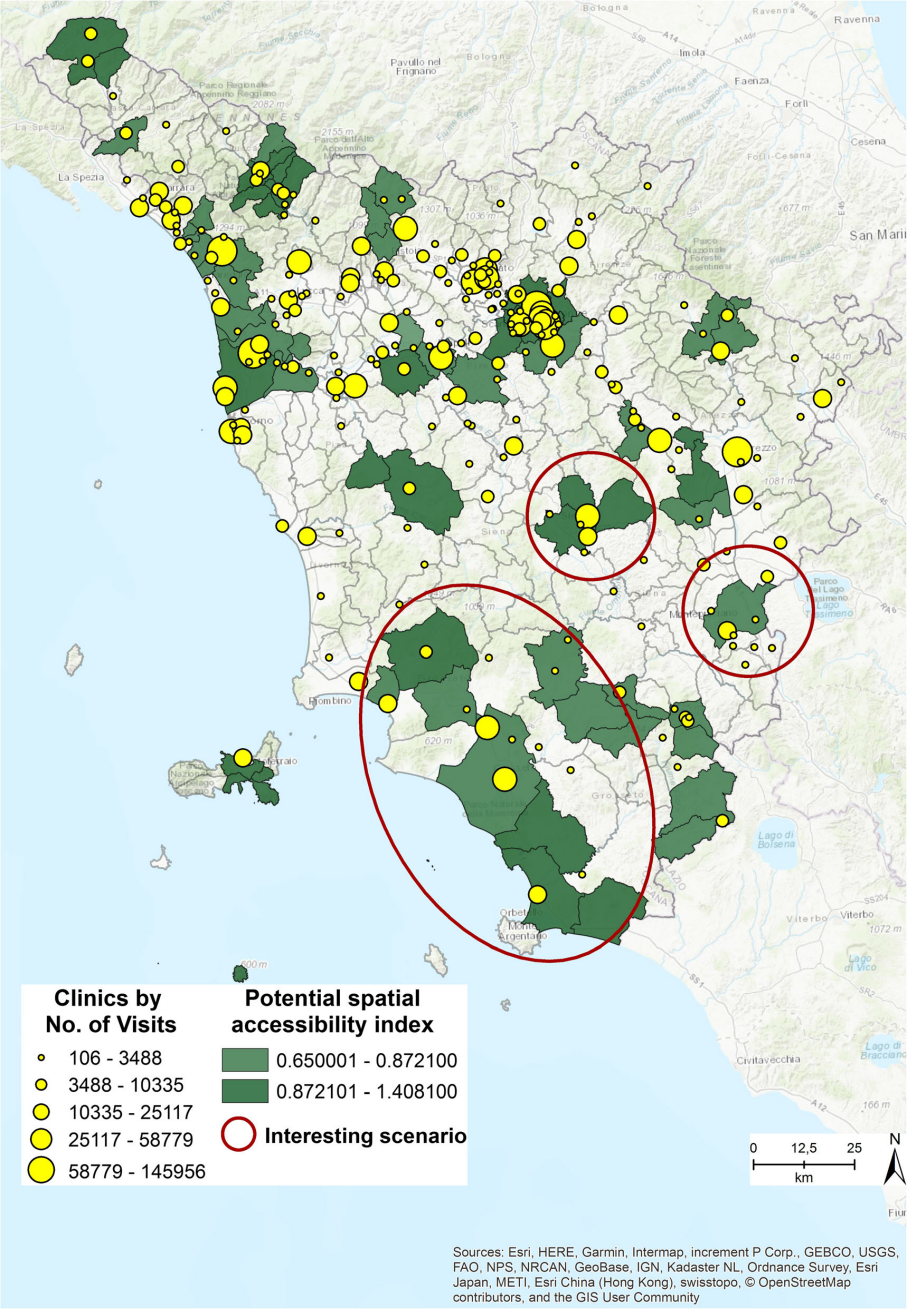
Municipalities characterized by a potential spatial accessibility index > = .65 [The figure is created by the authors and is not taken from other sources]

Figure 4 shows the distribution of the outpatient clinics by number of visits provided, and the municipalities with a spatial accessibility index higher than .65. Out of the total, 17 municipalities are characterized by a high value index. The scenario simulation focuses on three interesting cases, highlighted with red circles, which can provide evidence to decision makers when evaluating the outpatient delivery system. The cases present solutions in which the outpatient clinics could be for example merged or closed, thus increasing efficiency, delivering more visits, and at the same time maintaining an equitable accessibility level.

**Fig. 5:**
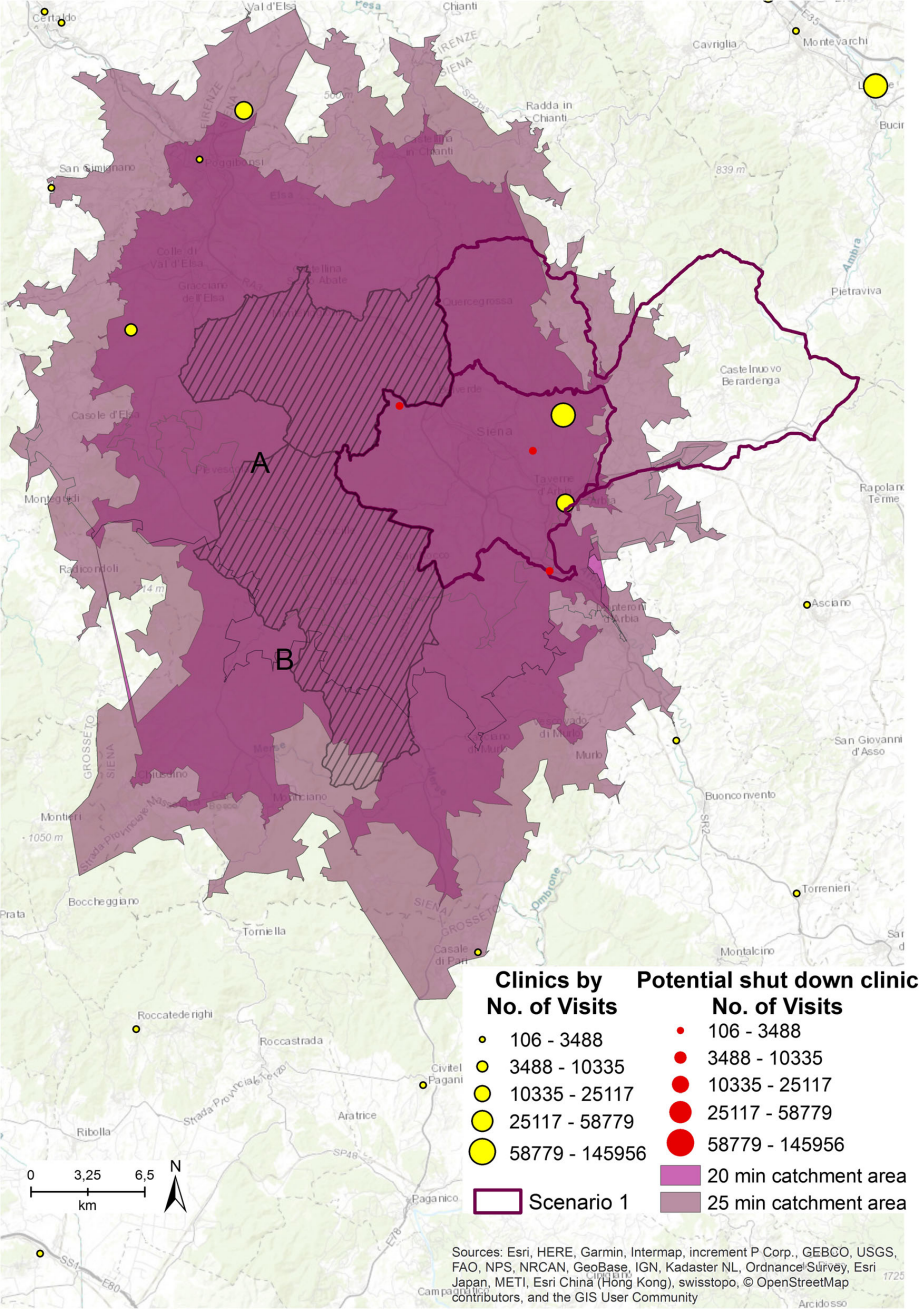
First scenario [The figure is created by the authors and is not taken from other sources]

Figure 5 illustrates the first scenario. Two municipalities and three outpatient clinics (represented in red and sized by the number of visits provided) that belong to the first quintile class are involved in the potential closure simulation.

If these three clinics were shut down, the residents of the two municipalities highlighted in Fig. 5 by the striped cover and named A and B, next to the two with the high accessibility values (highlighted by the purple border as Scenario 1), will no longer have access to any clinics within 15 min.

The study does not consider emergency services but focuses only on planned specialized patient visits. As urgent access to clinics is not considered, 5 and 10 min have been added to the catchment area simulation. Catchment areas of 20 (colored in orchid purple) and 25 (colored in dark cabernet purple) driving minutes from both the A and B centroid municipalities were run, to assess how accessibility to the clinics can change if the driving area is expanded. Within 20 min, residents of municipality A can have access to 4 clinics, while residents of municipality B can have access to 1 clinic. Within 25 min, residents of A can have access to 6 clinics and residents of B can have access to 4. As residents of A and B must drive a maximum of 10 min more to have access to specialized visits, we hypothesize shutting down the 3 clinics.

**Fig. 6:**
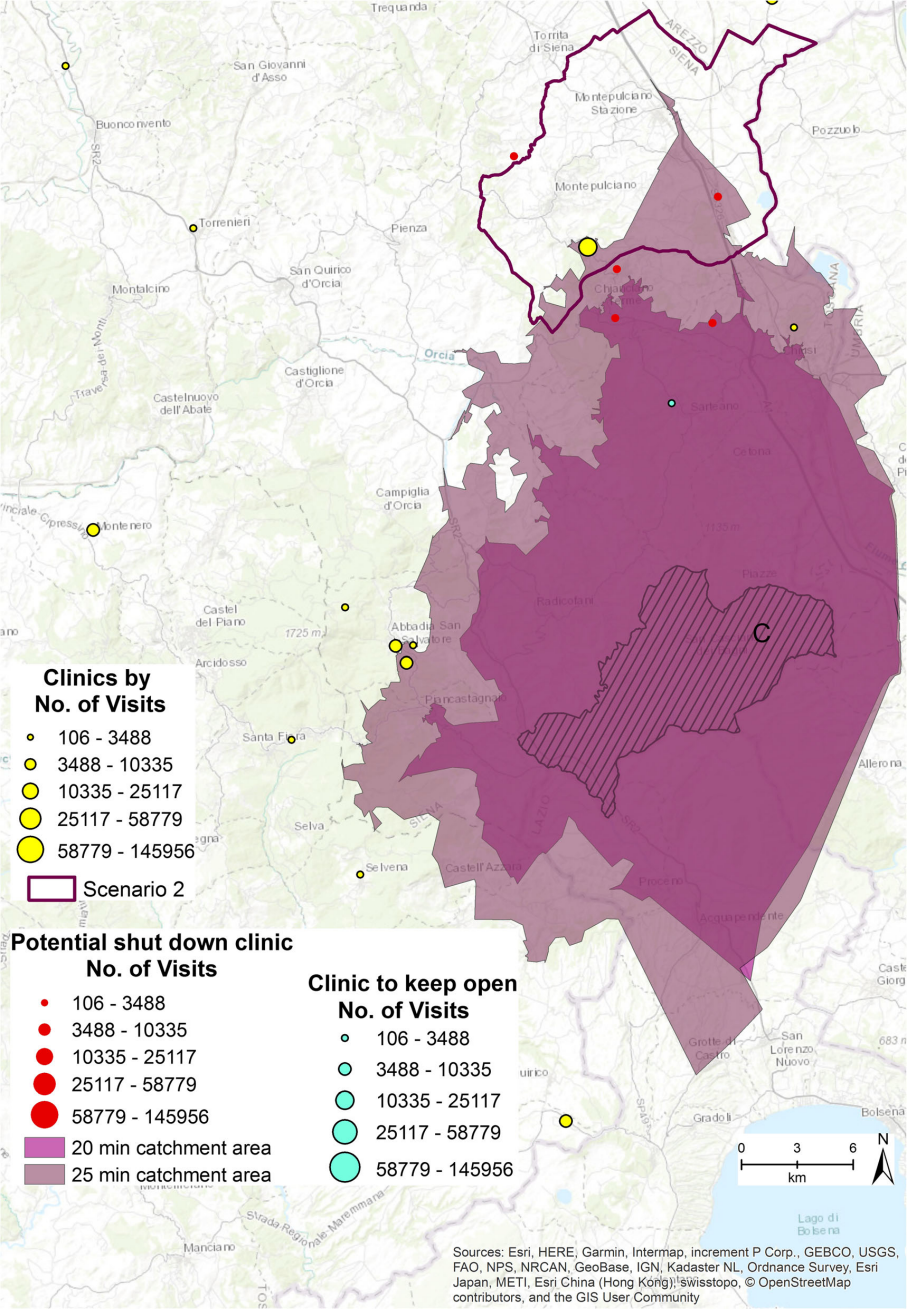
Second scenario [The figure is created by the authors and is not taken from other sources]

The second scenario is presented in Fig. 6. One municipality is characterized by high accessibility value, labeled as Scenario 2. Six clinics (five represented in red, one in light blue and sized by the number of visits provided) that belong to the first quintile class and have delivered 1133 specialized visits in 2015 are involved in the potential closure simulation.

If the six clinics were shut down, the residents of municipality C, (represented in Fig. 6 by the stripes), can no longer have access to any clinic within 15 min. Additionally, 20 (colored orchid purple) and 25 min (colored dark cabernet purple) driving catchment area analyses were run from the centroid of municipality C to establish how many clinics the residents have access to. They still have no access to any clinics within 20 min, while if they drove for 25 min they can access 5 outpatient clinics.

If one of the six clinics (colored light blue in Fig. 6) involved in the simulation remains open, residents of municipality C have access to at least one specialized clinic within 20 min. This solution guarantees more equitable access to the specialized health services of the area.

**Fig. 7:**
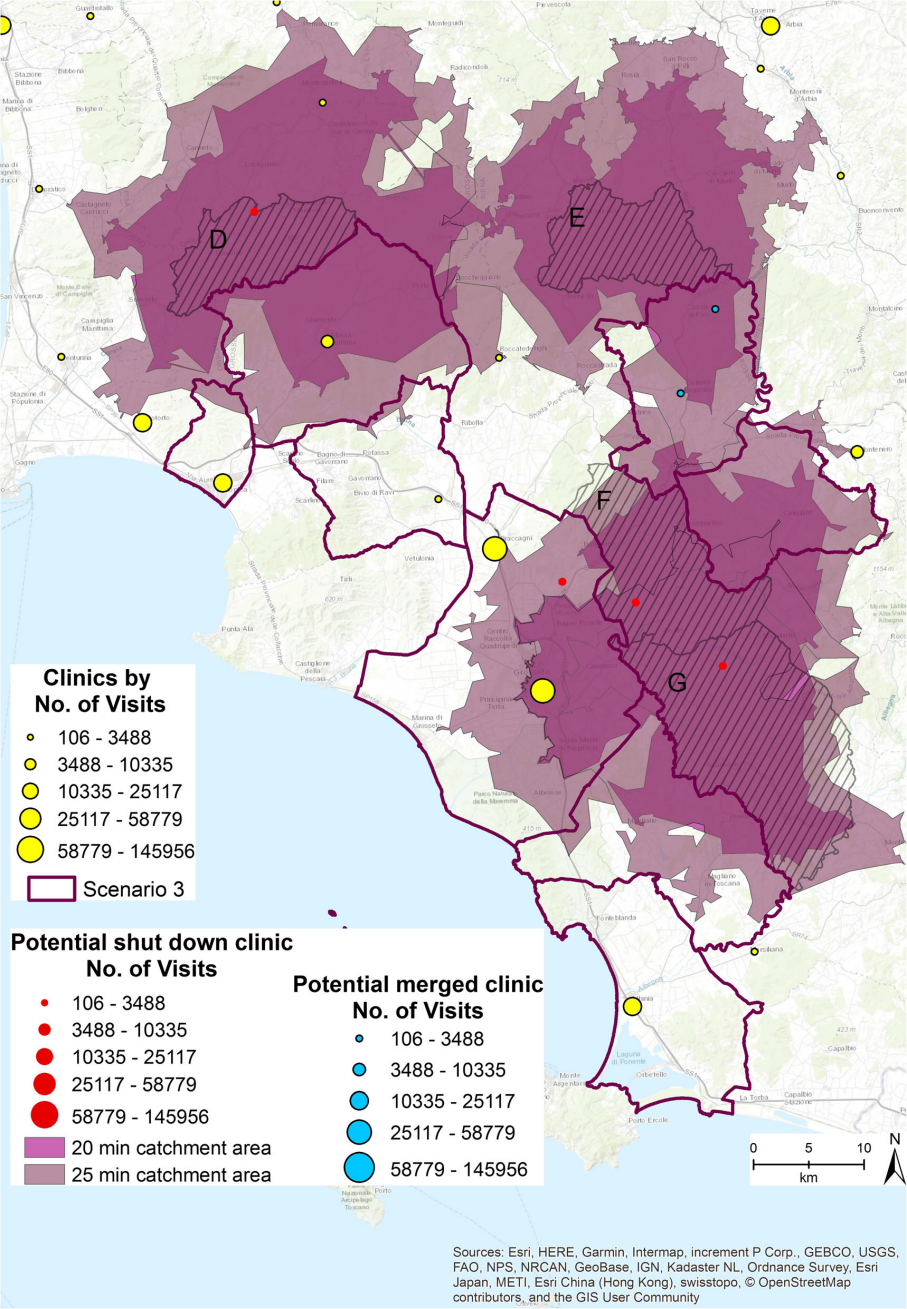
Third scenario [The figure is created by the authors and is not taken from other sources]

Figure 7 shows Scenario 3. Eight municipalities and six outpatient clinics (1403 specialized visits provided in 2015) are involved in the simulation. The clinics are highlighted in Fig. 7 in red and blue. If all the 6 clinics were shut down the residents of municipalities D, E, F, and G, highlighted in Fig. 7 by the stripes and the specific labels, no longer have access to any clinics within 15 min. The 20 (colored orchid purple) and 25 min (colored dark cabernet purple) catchment areas have been computed. Within 20 min’ drive, residents of municipality D have access to 2 clinics, those in municipality E still have no access, those in municipality F have access to 1 clinic, and those in municipality G do not have access to any clinics. Within 25 min, residents of municipality D have access to 3 outpatient clinics, those in municipality E still have no access, those in municipality F have access to 1 clinic, and those in municipality G have access to 1 clinic.

Given these preliminary results, a different managerial strategy is required in this area. The clinics colored blue in Fig. 7 have been merged into 1, allowing all the 4 municipalities D, E, F, and G to have access to at least 1 clinic within 25 min’ drive.

The merging strategy fits well with the aim of reducing inefficiencies and over supply services, while maintaining equitable access to specialized clinics.

**Fig. 8:**
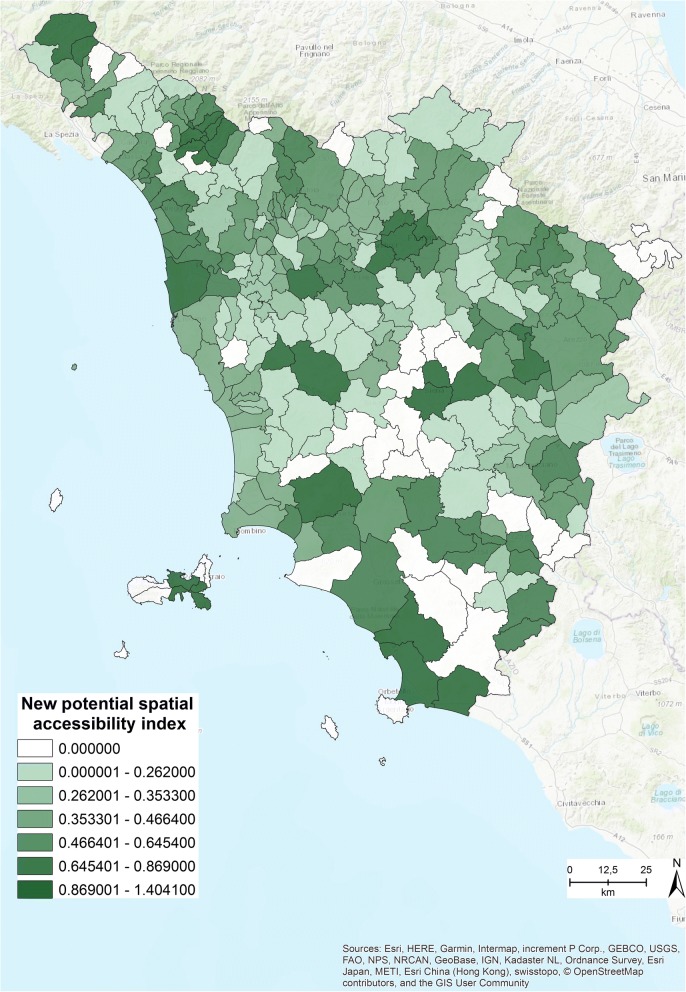
New potential spatial accessibility index within 15 min [The figure is created by the authors and is not taken from other sources]

The closure simulation revealed that 39 clinics can be shut down, thus reducing the total from 246 to 207. The new spatial accessibility index was run based on this new number of clinics, and the results are presented in Fig. 8.

Comparing Fig. 3 with Fig. 8 allows us to easily identify the differences between the original accessibility index (Fig. 3) and the new index (Fig. 8). The closure simulation revealed that most of the municipalities are covered within 15 min, and only in a few cases must residents travel for a maximum of 25 min to access at least 1 of the specialized outpatient clinics.

## Discussion

The aim of this study is to increase awareness of the importance of implementing a comprehensive geographical health information infrastructure, to support policy makers and healthcare managers with evidence-based solutions for the provision of health care services related to the needs. The study provides possible solutions to assist the planning and restructuring of regional health care services through an analysis of accessibility to outpatient clinics. Ensuring that resources are used effectively and efficiently when complying with regional and national regulations is essential, while also taking into account the population’s needs (demand for services) [32].

Using an integrated framework of geographical and administrative healthcare data enabled the identification of the municipalities that need to reorganize their delivery of outpatient services more efficiently by rethinking new clinic allocation, shutting down, or merging clinics. In particular, through the analyzed scenarios, the study identifies where delivery oversupply occurs in the current clinic distribution, which can be interpreted as an overestimation of the real needs of the population.

Seventeen areas with a high potential spatial accessibility index were identified. The analyses revealed that in 7 of these, residents could not access any clinics within 15 min’ drive time, which was set as the minimum travel time distance.

In these seven areas, few clinics belong to the first quintile class of volumes (those with low volumes of consultations per year), and thus could be closed. Most of these clinics need to stay open to guarantee equitable access. In the remaining 10 municipalities, all the clinics that belong to the first quintile can be reorganized, through a strategic shut down or merging solution. Out of the original 246 outpatient clinics, 38 could be directly shut down and 2 clinics could be merged. The total number of clinics that the simulation allows to close is 39, guaranteeing the population accessibility to outpatient services in a maximum of 15 to 25 min.

The methodological framework presented can be a valuable instrument for healthcare planners, as it can enable them to understand how outpatient services are organized and distributed. The study suggests ways to improve the efficiency of the delivery network while preserving equitable access.

The research has some limitations. The study had to deal with the problem of population data aggregation. To better shape the spatial distribution of the population, a dasymetric model has been applied to establish the centroids of each municipality, aggregating the data of the population. The dasymetric model helped to reduce the distance errors that can originate from the use of aggregated data. As in all studies that use aggregated data, there may be a statistical bias as a result of the modifiable areal unit problem (MAUP). To minimize the effects of MAUP, the smallest administrative units possible were used for the analyses [35, 36].

In addition, the study did not take into account flows of patients that immigrate into Tuscany. This phenomenon can seriously affect the volumes of the clinics at regional borders. However, not considering external residents’ flows does not affect the spatial accessibility in terms of equitable access to the outpatient clinics among Tuscan residents.

The measurement of patients’ specialized health care service needs is an important aspect of this study. The number of visits was used as a proxy for the level of need, although it is acknowledged that there is no direct correspondence between need and use [33, 34]. Scholars have proposed healthcare need indices [35], but the lack of an approved health service utilization rate has been identified as a research gap, highlighting the importance of this analysis [33].

Despite these limitations, the study underlines the importance of the integration of geographical components into healthcare administrative data to determine the provision and location of health care services in relation to needs. Indeed, the simulation analysis enables the identification of potential improvements in health care service delivery efficiency and value in health care, through reducing unnecessary cost and waste while maintaining or improving quality and access.

Additionally, this is the first study to our knowledge that focuses on the development of a potential spatial accessibility index in the Italian context, and that provides scenarios for describing and understanding the spatial organization of health care.

## Conclusions

A series of health care reforms based on strategic resource allocation have been implemented in Italy, to develop a more efficient delivery system. This system should also reflect the real needs of the population and guarantee equal access to quality care for all. The study originates from the view that to accomplish these tasks any reform must be grounded on solid evidence-based assessments. In addition, the assumption that geographical analyses can be useful in simulating and visualizing the effects of policies and thus legitimizing the introduction of new managerial strategies Informed the research question “How can a GIS support the health care management in the reorganization, redesign and planning of health care services?”

The research has demonstrated the importance of geographical instruments as convenient and easily readable tools in supporting the health care sector. The developed framework addresses the research question, demonstrating the benefits of GIS approaches within decision-making processes, and thus can help health care planners assess the effects of health policy from a geographical perspective [24, 30].

Based on the results, the authors recommend the use of geographical analyses in tackling the challenges of health reform, and to support decision-making processes. In 2015, Neutens highlighted how GIS was becoming increasingly recognized as an important and valuable instrument in mapping the spatial distribution of health care needs. Neutens also showed how GIS tools can be used in monitoring and evaluating the socio-spatial impacts of health policies, which can redress the balance of inequitable access and the disparities of health outcomes [21, 36], and thus gives greater theoretical foundation to this research.

## Abbreviations

GIS: Geographic Information System
WHO: World Health Organization
2SFCA: Two-step Floating Catchment Area.
ER: Emergency Room
MAUP: Modifiable Areal Unit Problem
